# Supplementary material for: Confidence intervals by constrained optimization—An algorithm and software package for practical identifiability analysis in systems biology
Source: PLoS Comput Biol. 2020 Dec 21;16(12):e1008495. doi: 10.1371/journal.pcbi.1008495 (PMC7785248; doi:10.1371/journal.pcbi.1008495)
Supplement: Attachment — Submitted filename: Letter to reviewers.docx [file pcbi.1008495.s001.docx]

Dear reviewers,

Thanks a lot for your interest and careful attention to this work. We appreciate your feedback and we have tried to address all drawbacks of the manuscript, which you have pointed out, namely:

**Reviewer #1:**

*I think that section 3.4 is unnecessary. I think it's fairly clear that this kind of numerical method needs to be computed on some finite support so practically all determinations are going to be made in some box. I don't think that more than a sentence or a paragraph is really required to get that point across.*

- We agree that section 3.4 had too many self-evident and unnecessary details. We have decided to shorten it and combine it with section 3.5. Nevertheless, we suppose that the explanation of scan bounds and their relation to identifiability is still necessary because: (1) it fills the gap between the definition of identifiability analysis and its practical usage; (2) explains how the termination criteria for CICO method work. See lines 252-278.

*The paper itself doesn't seem to have a lot of the validation. One example is used as validation, but the paper needs more.*

- We decided to include the STAT5 Dimerization model (one of the benchmark models for dMod software package) to the Validation part of the manuscript to broadly demonstrate the validity of the method. See section 3.7. We have also added this model to the GitHub repository.

*What they established was "structural efficiency", i.e. efficiency in terms of likelihood function evaluations. But it would've been nice to also see "practical efficiency", i.e. raw timings for the MATLAB method and Julia and Python implementation of the new methods, and use this to demonstrate a clear orders of magnitude actual performance improvement.*

- Timings have been added to the comparison tables to demonstrate “practical” efficiency of the method. See the updated tables 1 and 2.

**Reviewer #2**

*Equation 7: precise the values for j, to make clearer the fact that this is a system of more than 2 equations.*

- Necessary corrections were made to the index notation of the system of Equations 7 and 8. See lines 199-203. We have also made all vector values **bold** to avoid the confusion.

*Equation 8: it is not obvious how the authors transformed system (7) into system (8). More explanations are needed here since this is key to understand the algorithm. Are the systems strictly equivalent? In the definition of c, the authors need to precise the position of the “1” in the vector.*

- The section “3.2 Method Rationale” was rewritten to clarify the transformation from the system of equations 7 to 8, point out important assumptions and address the question of equivalency of the systems. Additional assumptions implied by CICO method are discussed in section 3.2, lines 224-229

*The authors claim in the Abstract and Introduction that their method provides more accurate estimation of confidence Interval bounds. However, this is not demonstrated in the article, neither theoretically, nor computationally (On the opposite, they do provide some evidence of the lower computational cost of their algorithm compared to existing ones). Please either add the corresponding evidence or modify the text*.

- We agree with this point. Practically we demonstrate the speed of computations. Regarding the accuracy, we decided to reformulate this statement as follows. “The numerical implementation of the algorithm includes settings to control the accuracy of confidence intervals estimates” which we believe is the benefit of the approach in comparison with other methods (see lines 29-32, 96-98). The algorithm proposes the users to set up tolerance, which is utilized by the optimizer as the termination tolerance.

We have highlighted all the updates in the text of the manuscript. Hope these changes will make the manuscript more clear. We will be happy to respond to other questions if they arise.

Kind regards,

Ivan Borisov, Evgeny Metelkin

InSysBio, LLC
